# Supplementary material for: Enhancement of RecET-mediated in vivo linear DNA assembly by a xonA mutation
Source: PLoS One. 2026 Apr 3;21(4):e0344368. doi: 10.1371/journal.pone.0344368 (PMC13048471; doi:10.1371/journal.pone.0344368)
Supplement: S1 Text — (PDF) [file pone.0344368.s001.pdf]

Supporting information

Enhancement of RecET-mediated *in vivo* linear DNA assembly by a *xonA* mutation

James A. Sawitzke[1,2,#a*], Nina Costantino[2,3,#a], Adriana Castillo Caballero[1], Ellen Hutchinson[2,#b], Alessandro Barenghi[1], Lynn C. Thomason[2,4,#c], & Donald L. Court[2,4,#a]

[1] Genetic & Viral Engineering Facility, Epigenetics and Neurobiology Unit, European Molecular Biology Laboratory, Monterotondo (RM), Italy

[2]Gene Regulation and Chromosome Biology Laboratory, Frederick National Laboratory for Cancer Research, National Cancer Institute, Frederick, Maryland, United States of America

[3]RNA Biology Laboratory, Frederick National Laboratory for Cancer Research, National Cancer Institute, Frederick, Maryland, United States of America

[4]Basic Science Program, Frederick National Laboratory for Cancer Research, Frederick, Maryland, United States of America

[#a]Current address: Frederick, Maryland, United States of America

[#b]Current address: Ascension Street Vincent Women and Infants Hospital, Indianapolis, Indiana, United States of America

[#c]Current address: Eugene, Oregon, United States of America

*corresponding author. jim.sawitzke@gmail.com (JAS)

Supporting Results and Discussion

Effect of *recJ* and *exoX* mutations on linear DNA assembly. In light of the possibility that host single-strand DNA exonucleases other than ExoI could also process linear DNA introduced by electroporation or formed during the recombination reaction, we asked whether the removal of two other *E. coli* single-strand DNA exonucleases affected the frequency of recombinant recovery for assembly of the pBR-*lacZ* plasmid. Strain NC558 is deleted for *recJ*, which encodes the RecJ protein, a processive 5'→3' ssDNA exonuclease [1]. When NC558 was tested for assembly of six fragments, we observed an average frequency of $1x10^2$ $Amp^R/10^8$ viable cells, similar to that seen in LT1795, the *xonA*$^+$ strain, indicating that the *recJ* mutation had no effect. 80% of the recombinant colonies obtained from the *recJ* mutant cells were blue on X-gal indicator agar, indicating that assembly was accurate. 25 white colonies (indicating defects in the *lacZ* gene) were analyzed by isolating their plasmid DNA. Restriction analysis demonstrated that nearly half (11/25) of the *lacZ* mutant plasmids were not assembled correctly in the *recJ* mutant background, a similar percentage to that seen for LT1795, the *recJ*$^+$ strain.

E. coli ExoX is a distributive 3'→5' exonuclease active on both ss- and dsDNA [2]. A strain deleted for the *exoX* gene, NC567, was tested for the ability to assemble six linear DNAs. The average recombination frequency observed with this strain was $5x10^1 Amp^R/10^8$ viable cells, again similar to both the wildtype LT1795 strain and the *recJ* mutant. A total of 9 Amp^R colonies were recovered and found to be blue on X-gal indicator agar. The *exoX* and *recJ* mutant results suggest that neither of these exonucleases plays a role in the fragment assembly reaction.

Supplemental Methods for experiments described in this paper.

Preparing linear DNA molecules for use in assembly. When plasmid DNA was used for PCR, ~100 ng of the plasmid was first digested with two different restriction enzymes, producing a linear DNA. The digest preparation was diluted 1000-fold and used as a template for PCR with primers that created fragments with 50bp terminal overlap (unless otherwise noted). In all cases, individual DNA fragments generated by PCR were tested before use in DNA assembly by introduction into competent DH5α cells by electroporation. These control transformations were plated on L+Amp agar. An absence of Amp^R colonies indicates an absence of any residual uncut plasmid DNA (i.e., confirmed complete digestion) from that used as template.

Linearized pLT59 was used as the PCR template for all fragments used in 2-way and 3-way recombination experiments. The linear DNAs generated with PCR contain a partial (nonfunctional) *ori* and *kan* gene; thus, only precise joining *in vivo* will result in an *ori*$^+$ recombinant that can replicate and confer kanamycin (Kan) resistance. Assembly of pLT59 from two DNA fragments (1548 and 2490 bp in length) is shown in Figure 1B; assembly of the same plasmid from three DNA fragments (1296, 1327, and 1465 bp in length) is shown in S1 Fig.

We designed the plasmid designated pBR-*lacZ* to further test the assembly of six fragments. In pBR-*lacZ*, the tetracycline resistance gene, *tet*, of pBR322 is replaced with the *E. coli lacZ* gene, retaining the *tet* regulatory elements (see Figure 6A). To assemble the pBR-*lacZ* plasmid backbone, three separate PCR products were generated, using linearized pBR322 as a template. Both the plasmid *ori* and the *bla* gene are split between two fragments. All three fragments were

electroporated separately into cells to ensure that no intact pBR322 plasmid was present. The 3075bp *lacZ* gene is assembled from three separate linear DNA fragments. These DNAs were either generated by colony PCR or synthesized as gBlocks (IDT). All six fragments must be accurately and precisely joined in correct order to result in a plasmid with an intact (a) *ori*, (b) *bla* gene, and (c) *lacZ* gene. Amp-resistant colonies (Amp^R) will only be recovered if both the *ori* and *bla* genes are functional. The *lacZ* gene encoding β-galactosidase allows a blue/white colony screen for fidelity of DNA joining of the *lacZ* fragments; only blue colonies express a functional *lacZ* gene. Fragment sizes were 1102, 1030, 1154, 1030, 1040, and 922bp in length.

To create linear DNA fragments of decreasing homology lengths, the oligos used to amplify the linear DNA by PCR were shortened by the appropriate number of bases on their 5' end, thus maintaining the same fragment with a shorter homology region.

General procedures for linear DNA assembly. The following guidelines are given for cloning genes of interest into plasmid backbones. Although expression vectors with multiple cloning sites are useful for this, any plasmid backbone can be used. See Thomason *et al.* [3] for detailed recombineering methods.

Care of strain NC553: The bacterial strain NC553 is temperature-sensitive since it contains a defective λ prophage with the *recET* and λ *gam* recombination genes under control of the *c*I*857* gene that encodes a temperature-sensitive repressor. Cells must always be grown at 30-32 °C. Propagation at higher temperatures will yield poor growth and give rise to mutants unable to express the recombination functions. Upon receiving the strain, a glycerol stock should be made from bacteria grown on either LB solid agar or in LB broth. When growing cells for fragment joining, always grow the strain at 30-32 °C until the 42 °C heat induction step for expression of the recombination functions. NC553 contains no plasmid or drug markers, making it possible to assemble plasmids with different DNA replication origins and select for a wide range of drug resistances in this background.

Preparation of vector: Digest 100 ng of the vector, preferably with two unique restriction enzymes (made easier with a multicloning site) to completely linearize the vector. Clean the digest using a commercial kit (such as Qiagen), eluting in 50 µL of dH_2O or elution buffer supplied with the kit. Overhangs left by restriction enzymes are not a concern, and like others [4] we have found that target homologies that are 10 bases or more internal to the cut site still yield high frequencies of correct fragment joining, with terminal non-homologies removed during the recombination process. If there are no appropriate restriction sites, it is possible to PCR amplify the plasmid origin of choice.

In all cases, the linear vector preparation must be tested to ensure complete restriction enzyme digestion by introducing 1µl of the purified prep into electrocompetent cells (such as commercial DH5α) by electroporation. After a 2-hour outgrowth, spread 100 µl of cells on a solid agar LB plate containing an appropriate concentration of drug corresponding to the antibiotic resistance marker on the plasmid backbone. The resulting colony count should be <10 colony-forming units (cfu). If large numbers of drug-resistant cfu are found, plasmid restriction must be improved until the background count is lowered; an alternative is to purify the linear fragment from an agarose gel. Do not omit this control of testing the vector alone for complete

linearization, since incomplete cutting will give rise to a high background of colonies containing only the vector, which will swamp out the yield of assembled plasmids containing your desired fragment(s). Note that simply visualizing the digested vector on an agarose gel to look for a supercoiled plasmid is not an adequate control. Trace amounts of uncut plasmid may be invisible on the gel but can still give substantial numbers of drug-resistant colonies after transformation by electroporation.

Preparation of fragments: Other DNA fragments to be incorporated onto the plasmid backbone should be designed to contain appropriate 30-50 bp homologies at each end. We routinely use 50 bp of overlapping homology. Colony PCR [3] can be used to amplify genes from the *E. coli* chromosome or a BAC. A plasmid can be used as a template if care is taken to completely linearize it, especially if it contains the same drug resistance you will be selecting for in your assembly product. As with the vector, these DNAs should be purified to remove salts and visualized by agarose gel electrophoresis to ensure the correct size fragments. It is not always necessary to quantitate and dilute this DNA, as long as adequate yields are obtained, we routinely simply use 1 µl of the cleaned PCR fragment. Synthetic dsDNA (i.e., gBlocks from IDT) is reconstituted at 100 ng/µl, and 1 µl is used per recombination.

Growth and induction: Details of cell preparation for electroporation and recombinant proficient cells are given in Thomason et al. [3]. Briefly, an overnight culture is diluted into fresh L broth and grown at 32 °C to log phase. At this point, the culture is divided and half of the cells are shifted to 42 °C for 15min to induce expression of the *recET* genes, with the remaining culture remaining at 32 °C. This 42 °C temperature induction requires a shaking water bath for efficient heat transfer (see https://redrecombineering.ncifcrf.gov/). The short induction time is critical as prolonged expression of the recombinations functions is toxic. After 15 min, both flasks are rapidly chilled in an ice water slurry. The cells are then made electrocompetent. We have not tried using calcium-competent cells for linear DNA assembly.

Electroporation and outgrowth: The DNAs to be assembled are introduced into electrocompetent and RecET recombination proficient (induced at 42 °C) cells in which recombinants are generated. Uninduced (32 °C) cells are used as a control to check for the presence of uncut vector [3]. After a 2 hr outgrowth in 1 ml LB broth at low temperature (30-32 °C), five consecutive 10-fold serial dilutions of the culture are made into sterile M9 salts or Tris-Magnesium (TM) [3] or LB broth. From the last two dilutions (10^{-4} and 10^{-5}), 100 µl of the mixture is spread on LB agar to determine total viable cell counts. From the undiluted culture and the 10^{-1} and 10^{-2} dilutions, 100 µl is spread on LB agar containing the appropriate drug to select for intact plasmids. Plates are incubated at 30-32 °C overnight (or two days if needed) and scored on LB agar with the appropriate drug for bacterial colonies. Few if any colonies should be found with the uninduced 32 °C cultures. Such drug-resistant colonies from the uninduced culture indicate an uncut plasmid background. The recombinant frequency is calculated using titers from the induced (42 °C) RecET plates as: (drug-resistant colonies/total viable colonies)10^8.

Identifying recombinant clones: Purify 12 colonies picked from the drug plates for the induced 42 °C cultures onto the same solid medium and incubate these plates at 30-32 °C. Incubating the purification plates at higher temperatures will induce the RecET recombination functions and

(especially *gam*) kill the cells. These purified isolates can be screened for plasmid assembly either with colony PCR or by isolating DNA using a miniprep. For the former approach, perform colony PCR [3] on the 12 purified isolates to confirm recombinant junctions and screen for desired insertions. The PCR primers should be designed to span unique junctions joined together during assembly, to confirm assembly of the linear DNAs into plasmids. For smaller inserts, primers can be designed to span the entire region. These PCR products can then be directly sequenced. Alternatively, the purified colonies can be used to start overnight cultures for plasmid DNA minipreps for further analysis by restriction enzyme digestion, agarose gel analysis, and sequencing. We strongly recommend that all insertions be sequenced in their entirety, as well as recombinant junctions, to ensure that correct clones are identified. As noted in this paper, mistakes in inserts usually arise from either PCR or gBlock synthesis. We also note that Lac^+ (blue) isolates were sequenced, and even though the LacZ protein was functional, mutations resulting in amino acid changes were found. Of the seven Lac^+ plasmids sequenced from PCR assembly, all contained at least one mutation. Seven Lac^+ plasmids from gBlock assembly were also sequenced; four of these contained at least one mutation. On some occasions, we have seen frameshifts occurring at the beginning of an open reading frame when the gene to be cloned conferred toxicity on the bacterial cells.

Supporting References

[1] Lovett ST, Kolodner RD. Identification and purification of a single-stranded-DNA-specific exonuclease encoded by the *recJ* gene of *Escherichia coli*. Proc Natl Acad Sci U S A. 1989;86:2627-31.

[2] Viswanathan M, Lovett ST. Exonuclease X of Escherichia coli. A novel 3'-5' DNase and DnaQ superfamily member involved in DNA repair. J Biol Chem. 1999;274:30094-100.

[3] Thomason LC, Sawitzke JA, Li X, Costantino N, Court DL. Recombineering: genetic engineering in bacteria using homologous recombination. Curr Protoc Mol Biol. 2014;106:1.16.1-39.

[4] Fu J, Bian X, Hu S, Wang H, Huang F, Seibert PM, et al. Full-length RecE enhances linear-linear homologous recombination and facilitates direct cloning for bioprospecting. Nat Biotechnol. 2012;30:440-6.
