# Supplementary material for: Enhancement of RecET-mediated in vivo linear DNA assembly by a xonA mutation
Source: PLoS One. 2026 Apr 3;21(4):e0344368. doi: 10.1371/journal.pone.0344368 (PMC13048471; doi:10.1371/journal.pone.0344368)
Supplement: S1 Fig — Terminal homologies present in each fragment within the ori, bla, and kan genes are indicated by the single-stranded bases. One single-strand base indicates a homology of 50–60 bases. The data are shown in Fig 3 of the main paper. (PDF) [file pone.0344368.s002.pdf]

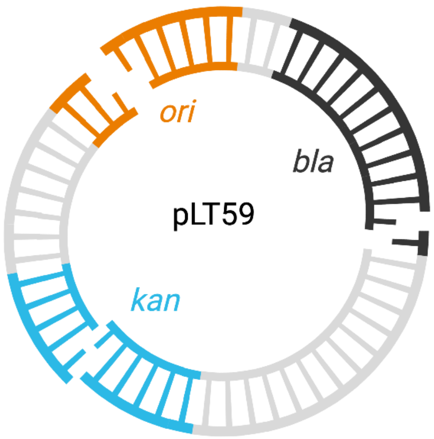

S1 Fig. DNA fragments used for the three-way assembly of pLT59. Terminal homologies present in each fragment within the *ori*, *bla*, and *kan* genes are indicated by the single-stranded bases. One single-strand base indicates a homology of 50-60 bases. The data are shown in Figure 3 of the main paper.

The same protocol was used to prepare six linear DNAs for recombination from plasmid pLT61 (see S2 Fig). This plasmid contains a chloramphenicol resistance cassette, *cat*, immediately flanked by its own promoter and transcription terminator inserted into the *kan* coding region. In this 6-way linear assembly, the *ori*, *bla,* and *cat* functional regions are each brought in by two separate PCRs. The linear DNAs used for assembly were 941, 766, 912, 875, 902, and 858 bp in length.
