# Supplementary material for: Enhancement of RecET-mediated in vivo linear DNA assembly by a xonA mutation
Source: PLoS One. 2026 Apr 3;21(4):e0344368. doi: 10.1371/journal.pone.0344368 (PMC13048471; doi:10.1371/journal.pone.0344368)
Supplement: S2 Fig — A. Six PCR fragments were used to assemble the circular plasmid pLT61. Of the six junctions, one is within the plasmid ori, a second is within the bla gene, and two more are within the chloramphenicol resistance gene, cat. B. Elimination of the host ExoI function (i.e., ΔxonA) increased the frequency of plasmid assembly ~1000 fold. When AmpR colonies from the RecET ΔxonA recombination were scored for CmR, 194/200 colonies were CmR, indicating accurate joining of the linear DNA segments. We anticipate that most of the CmS plasmids arose from PCR mistakes or primer synthesis mistakes, as found for the lacZ gene in other six-way assembly experiments (see Fig 6, main paper). (PDF) [file pone.0344368.s003.pdf]

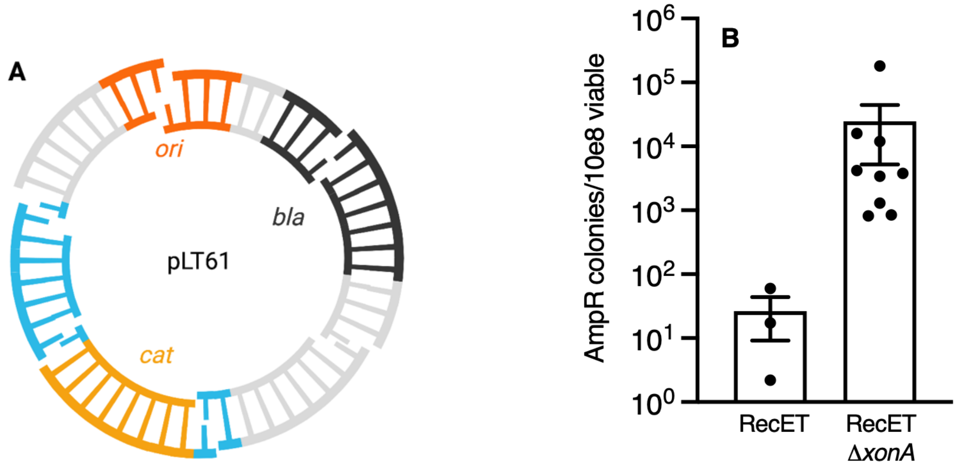

S2 Fig. Additional data for *in vivo* linear assembly from six linear dsDNA fragments. A. Six PCR fragments were used to assemble the circular plasmid pLT61. Of the six junctions, one is within the plasmid *ori*, a second is within the *bla* gene, and two more are within the chloramphenicol resistance gene, *cat*. **B.** Elimination of the host ExoI function (i.e., Δ*xonA*) increased the frequency of plasmid assembly ~1000 fold. When Amp^R colonies from the RecET Δ*xonA* recombination were scored for Cm^R, 194/200 colonies were Cm^R, indicating accurate joining of the linear DNA segments. We anticipate that most of the Cm^S plasmids arose from PCR mistakes or primer synthesis mistakes, as found for the *lacZ* gene in other six-way assembly experiments (see Figure 6, main paper).
