# Supplementary material for: Enhancement of RecET-mediated in vivo linear DNA assembly by a xonA mutation
Source: PLoS One. 2026 Apr 3;21(4):e0344368. doi: 10.1371/journal.pone.0344368 (PMC13048471; doi:10.1371/journal.pone.0344368)
Supplement: S3 Fig — The black bar with cyan segments represents a portion of the pBR-lacZ plasmid. The homology overlaps between fragments used for assembly are shown in cyan. The 3075 bp lacZ open reading frame (orf) is indicated by the grey arrow. The region from the promoter to the translational stop was sequenced; locations of observed mutations are indicated with vertical lines and a colored circle with a number, indicating the isolate number. If multiple mutations were found in an isolate, they are indicated with the same color and number. We did not find increased numbers of mutations in the overlapping homology region where the single-strand annealing occurs A. PCR products were used for assembly. A total of 20 isolates were sequenced and among these, 68 point mutations and 6 insertion or deletions were found. 9 candidates had mutations in the overlap homologies. B. gBlocks were used for assembly. A total of 20 isolates were sequenced and among these, 17 point mutations and 14 insertion or deletions were found. Only 3 isolates had any mutations in the overlap homologies. The 5 candidates not shown were isolates that had apparent synthesis errors containing small repeats. (PDF) [file pone.0344368.s004.pdf]

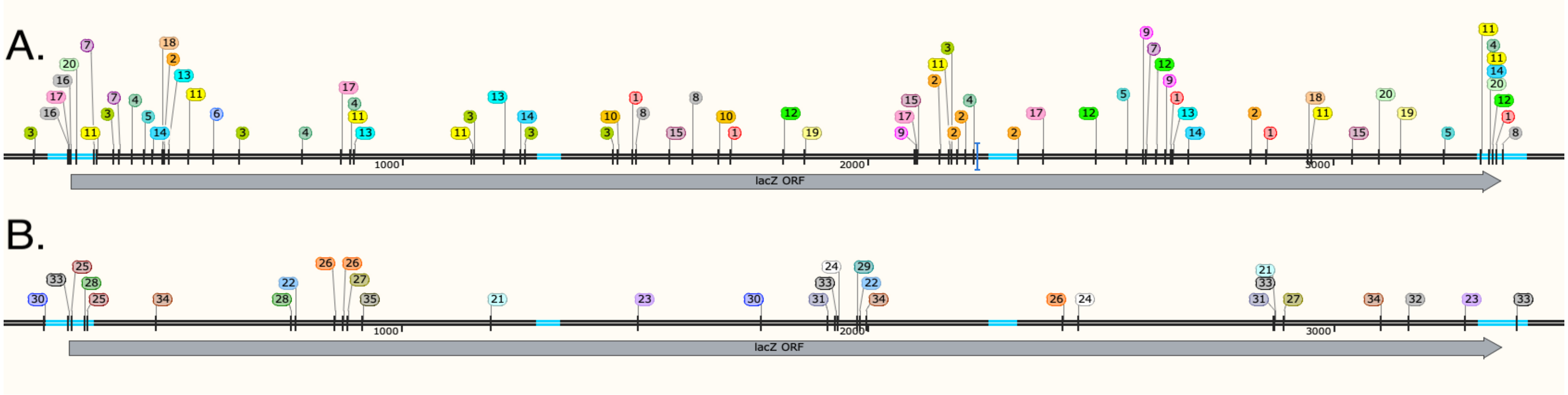

S3 Fig. Sequence analysis of white colonies from the pBR-*lacZ* assembly reactions.
The black bar with cyan segments represents a portion of the pBR-*lacZ* plasmid. The homology overlaps between fragments used for assembly are shown in cyan. The 3075bp *lacZ* open reading frame *(orf)* is indicated by the grey arrow. The region from the promoter to the translational stop was sequenced; locations of observed mutations are indicated with vertical lines and a colored circle with a number, indicating the isolate number. If multiple mutations were found in an isolate, they are indicated with the same color and number. We did not find increased numbers of mutations in the overlapping homology region where the single-strand annealing occurs A. PCR products were used for assembly. A total of 20 isolates were sequenced and among these, 68 point mutations and 6 insertion or deletions were found. 9 candidates had mutations in the overlap homologies. B. gBlocks were used for assembly. A total of 20 isolates were sequenced and among these, 17 point mutations and 14 insertion or deletions were found. Only 3 isolates had any mutations in the overlap homologies. The 5 candidates not shown were isolates that had apparent synthesis errors containing small repeats.
