# Supplementary material for: Enhancement of RecET-mediated in vivo linear DNA assembly by a xonA mutation
Source: PLoS One. 2026 Apr 3;21(4):e0344368. doi: 10.1371/journal.pone.0344368 (PMC13048471; doi:10.1371/journal.pone.0344368)
Supplement: S4 Fig — Terminal homologies present in each fragment within the ori, kan, lacZ, and bla genes are indicated by the single-strand bases. One single-strand base indicates a homology of 50 bases. Fragment #4 (indicated with a “4”) is always the smallest fragment and, in different experiments, was 100 bp, 400 bp, or even a 100-base oligo as shown in the data in Figure 7 of the main paper. The length of fragment 5 (within lacZ) varies according to the length of fragment 4; when fragment 4 is longer, fragment 5 is shorter, etc. (PDF) [file pone.0344368.s005.pdf]

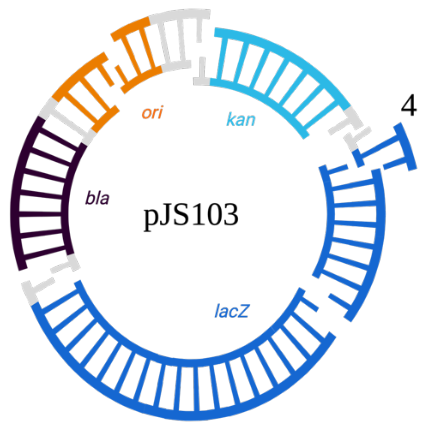

S4 Fig. DNA fragments used for the six-way assembly of pJS103. Terminal homologies present in each fragment within the *ori*, *kan, lacZ,* and *bla* genes are indicated by the single-strand bases. One single-strand base indicates a homology of 30 bases. Fragment #4 (indicated with a "4") is always the smallest fragment and, in different experiments, was 100 bp, 400 bp, or even a 100-base oligo as shown in the data in Figure 7 of the main paper. The length of fragment 5 (within *lacZ*) varies according to the length of fragment 4; when fragment 4 is longer, fragment 5 is shorter, etc.
