# Supplementary material for: Enhancement of RecET-mediated in vivo linear DNA assembly by a xonA mutation
Source: PLoS One. 2026 Apr 3;21(4):e0344368. doi: 10.1371/journal.pone.0344368 (PMC13048471; doi:10.1371/journal.pone.0344368)
Supplement: S1 Table — (PDF) [file pone.0344368.s006.pdf]

S1 Table. ***Escherichia coli*** **K-12 strains and plasmids for supporting information.**

**Strain Name**	**Relevant genotype**	**Source**
NC558	W3110 Δ*lacU169 galKTYR145UAG* Δ*recJ<>cat* λ*cI857* Δ(*cro-bioA*) (*int-cIII*)< >*gam recE recT*	this study
NC567	W3110 Δ*lacU169 galKTYR145UAG* Δ*exoX<>spec* λ*cI857* Δ(*cro-bioA*) (*int-cIII*)< >*gam recE recT*	this study
**Plasmid name**	**Relevant genotype**	**Source**
pLT61	pUC plasmid bla^+ cat^+	Thomason *et al.*[5]
