# Supplementary material for: Enhancement of RecET-mediated in vivo linear DNA assembly by a xonA mutation
Source: PLoS One. 2026 Apr 3;21(4):e0344368. doi: 10.1371/journal.pone.0344368 (PMC13048471; doi:10.1371/journal.pone.0344368)
Supplement: Attachment — Submitted filename: Response to reviewers.docx [file pone.0344368.s009.docx]

**January 26, 2026**

Dear Dr. Mienda,

Thank you for editing and sending out to review our manuscript entitled *“Enhancement of RecET‑mediated in vivo linear DNA assembly by a xonA mutation”.* We appreciate your comments and those of the reviewer and have addressed them as follows.

**Editor Comments:**

1., 2. We have made every effort to format the manuscript to the PLOS One guidelines.

3., 4. We have corrected our funding statement to say, “This work was supported, in part, by the Intramural Research Program of the National Institutes of Health, National Cancer Institute, Center for Cancer Research through D.L.C. This project has also been partly funded with federal funds from the National Cancer Institute, National Institutes of Health, under contract no. HHSN261200800001E through D.L.C. Funding was also provided by the European Molecular Biology Laboratory to J.A.S., A.C.C and A.B. There was no additional external funding received for this study. The funders had no role in the study design, data collection and analysis, decision to publish, or preparation of the manuscript.”

5. We have spelled out “EMBL” in that statement.

6. We have changed the statement, “Sequences of DNA oligos are available upon request” to “Sequences of DNA oligos are in S2 Table” and added S2 Table with their sequences.

7. On line 206 it used to say, “(data not shown)”. We have removed that phrase and made no further changes. The statement indicates the result as is.

8. Supporting information has been moved to separate files as requested. Also, the legends to the supporting files have been added to the main manuscript after the reference list.

10., 11. References have been checked and are accurate to our knowledge.

**Reviewer Comments:**

1. Figures: Checked that all references to figures are now correct. On line 143, deleted reference to “S3 Fig” as it indeed was incorrect.

Sincerely,

**James A. Sawitzke, Ph.D.**
[jim.sawitzke@gmail.com](mailto:jim.sawitzke@gmail.com)
